# Spontaneous Hip Dislocation Complicating the Management of Malignant Peripheral Nerve Sheath Tumor Arising Within a Plexiform Neurofibroma

**DOI:** 10.7759/cureus.16320

**Published:** 2021-07-11

**Authors:** Oliver D Mrowczynski, Monali Vasekar, Edward Fox, Kimberly Harbaugh, Dawit Aregawi, Colette Pameijer, Nicholas Zaorsky, Russell Payne, Elias Rizk

**Affiliations:** 1 Neurosurgery, Penn State Health Milton S. Hershey Medical Center, Hershey, USA; 2 Hematology, Penn State Health Milton S. Hershey Medical Center, Hershey, USA; 3 Orthopaedics, Penn State Health Milton S. Hershey Medical Center, Hershey, USA; 4 Neurosugery, Penn State Health Milton S. Hershey Medical Center, Hershey, USA; 5 Surgical Oncology, Penn State Health Milton S. Hershey Medical Center, Hershey, USA; 6 Radiation Oncology, Penn State Health Milton S. Hershey Medical Center, Hershey, USA

**Keywords:** mpnst, malignant peripheral nerve sheath tumor, nf1, neurofibromatosis type 1, plexiform neurofibroma, hip dislocation

## Abstract

Neurofibromatosis type 1 (NF1) is one of the most common inherited neurological disorders. It can cause plexiform neurofibromas, leading to diffuse enlargement of a nerve or nerves within the body. There are benign in general, however, can cause significant symptoms due to their size, including bony erosion, pain, and joint instability. Unfortunately, they also have the capacity to become malignant by internal transformation into a malignant peripheral nerve sheath tumor (MPNST). The case presented here is a 27-year-old male with NF1 that was followed for years with a pelvic girdle plexiform neurofibroma whose course was complicated by transformation to MPNST and a spontaneous hip dislocation. He underwent excision, Girdlestone procedure, chemotherapy, and radiation. Unfortunately, he subsequently developed lung metastases and is part of a clinical trial with an MDM2 inhibitor and pembrolizumab.

## Introduction

Neurofibromatosis type 1 (NF1) is a one of the most common inherited neurological disorders caused by a mutation in the neurofibromin gene [1-4]. Plexiform neurofibromas are unencapsulated nerve sheath tumors that cause diffuse enlargement of a nerve or nerves in a body region. They are found in 40-50% of NF1 patients [5-7]. They are benign but may cause symptoms due to pain, large size, surrounding tissue overgrowth, bony erosion, and joint instability. More ominously, plexiform neurofibromas may develop an area of malignant degeneration, a malignant peripheral nerve sheath tumor (MPNST) within them [6,8,9]. We present a case of a patient with NF1 who developed an MPNST in a pelvic girdle plexiform neurofibroma whose management was complicated by spontaneous hip disarticulation. Collaboration between the multidisciplinary NF and sarcoma teams helped optimize the management.

## Case presentation

**Figure 1:**
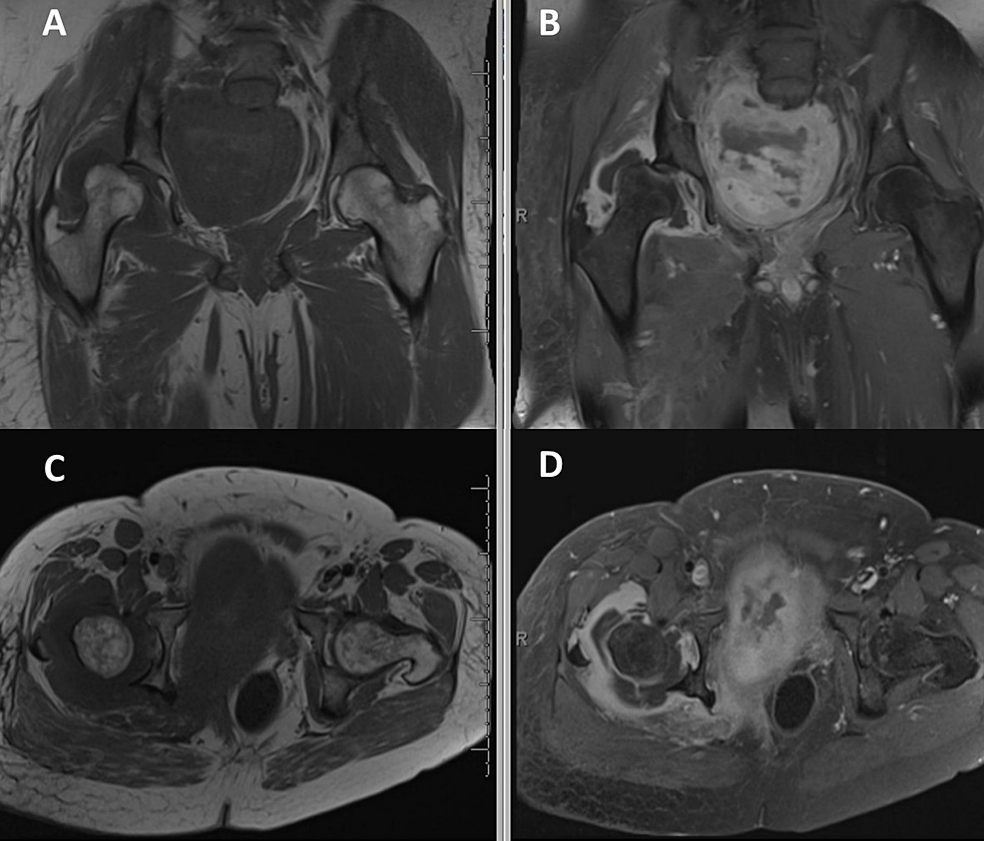
Pre and post-contrast T1-weighted pelvic MRI with coronal (A and B) and axial (C and D) images at the level of the hip. These images demonstrate an intra-articular/periarticular lesion that leads to a shallow acetabulum, resulting in the femoral head's dislocation.

This 27-year-old male NF1 patient was being followed in the NF clinic with known plexiform neurofibromas in the left submandibular region and right pelvis/hip girdle. He developed symptoms of lumbosacral plexopathy with right sciatica and ankle weakness and intermittent urinary retention. His previously stable pelvic tumor was reimaged and found to have increased in size from 15 × 12 cm^2^ to 19 × 15 cm^2^ (Figure 1).

**Figure 2:**
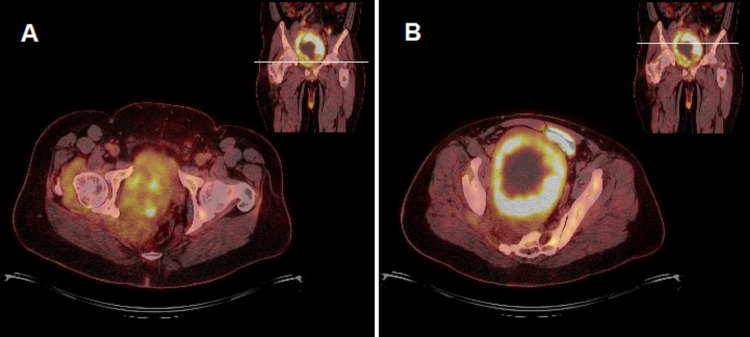
PET/CT axial images at the level of the hip (A) and the upper-middle portion of the pelvic mass (B). These images demonstrate areas of intense FDG avidity within the pelvic mass suggestive of malignant transformation. Much less avid and patchy FDG uptake of the neurofibroma can also be seen in the acetabulofemoral joint region.

On PET imaging, it had a central area of necrosis with a surrounding high SUV region suggestive of an MPNST (Figure 2).

**Figure 3:**
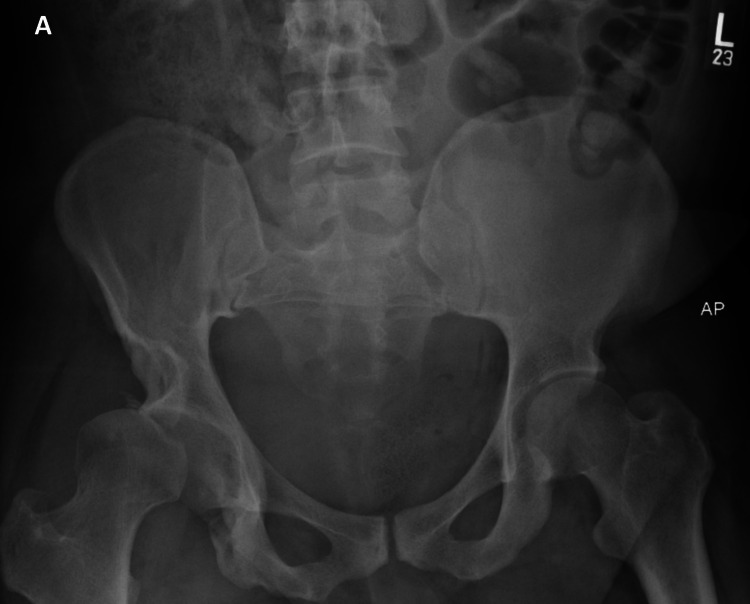
AP X-ray of the pelvis. This image shows superior subluxation of the right femoral head in the acetabulofemoral joint. Noted is the shallow nature of the right acetabulum compared to the contralateral side.

A biopsy confirmed a grade 3/3 MPNST. Given its size and location, neoadjuvant treatment was recommended prior to attempted surgical resection. Just prior to his scheduled admission for chemotherapy, he developed severe, acute right hip pain. Imaging revealed a fracture-dislocation of the hip (Figure 3). 

The initial concern was for a pathologic fracture, but on review of new and old imaging, it was felt to be due to chronic changes from the plexiform neurofibroma combined with hip girdle weakness from his MPNST-related lumbosacral plexopathy.

He was placed in tibial pin traction and subsequent hip abduction bracing. He was given two cycles of chemotherapy (doxorubicin/ifosfamide) followed by six weeks of inpatient radiation therapy (57.5 Gy, IMRT) to the pelvic region with sparing of the remainder of the plexiform neurofibroma. He had a complicated hospital course but eventually was discharged to a rehabilitation center. One month later, he underwent operative intervention targeting complete resection of the malignant, internal pelvic component of the tumor with sparing of the benign neurofibroma where it exited the pelvis. His postoperative course was complicated by a pelvic hematoma that resolved without intervention and worsening of his lumbosacral plexopathy with a complete foot drop. 

He was transferred back to the rehabilitation center but suffered multiple hip dislocations. This limited his recovery, and after discussion of treatment options, the patient underwent a Girdlestone procedure, which is an excision arthroplasty of the hip, two months after his MPNST resection. He recovered well and ultimately was able to ambulate with a walker. There was no evidence of malignancy in the hip specimen.

Unfortunately, 17 months after the Girdlestone procedure and 2 years after the initial MPNST diagnosis, he developed lung metastases. He failed treatment with a combination of gemcitabine and docetaxel with pancytopenia and the disease progressed. Currently, 2.5 years after the initial MPNST diagnosis, he is enrolled in a clinical trial with an MDM2 inhibitor and pembrolizumab.

## Discussion

Plexiform neurofibromas are found in 40-50% of NF1 patients. Although pathologically benign, they may be symptomatic, causing pain, neurologic deficits, tissue overgrowth, bony erosion, and articular instability [5-7]. Malignant degeneration within a plexiform neurofibroma with the formation of an MPNST is also possible, as was seen in our patient [6,8,9].

Our patient’s case was further complicated by hip disarticulation. Hip disarticulation related to plexiform neurofibroma has been reported previously [10-16]. We show a summary of the previous reports and their treatments in Table 1.

**Table 1:** Summary of the previous reports and their treatments

Study	Patient age	Genetics	Symptoms	Treatment
Rafaelian et al. [10]	27F	NF1	None found on incidental imaging	Observation
Galbraith et al. [11]	18F	NF1	Hip pain decreased range of motion	Skeletal traction
Guilleminet et al. [12]	Unable to obtain article			
Haga et al. [13]	6moM	NF1	Decreased ability to walk	Surgical resection and chemotherapy
Haga et al. [13]	3moF	NF1	Decreased ability to walk	Pavlik harness
Kumar et al. [14]	21F	NF1	Asymptomatic	Reduction under general anesthesia
Lachiewicz et al. [15]	37F	NF1	Left hip pain	Hip spica and hip abduction brace
Waheed et al. [16]	37M	NF1	Persistent left hip pain	Total hip arthroplasty

The imaging studies in our patient revealed a shallow acetabulum and dysplastic changes in the pelvis. These changes in association with the hip girdle and extremity weakness caused by the MPNST-induced lumbosacral plexopathy led to the fracture of the superior acetabulum and dislocation.

Recognition of these entities as the cause of the dislocation as opposed to a pathological fracture-dislocation was critical in this case. Direct MPNST involvement of the hip would have made this a non-surgical case, and the patient would have received palliative therapy alone. Differentiating the benign plexiform neurofibroma from MPNST in the radiated treatment field was also crucial as radiation of the benign tumor could lead to a secondary area of malignant degeneration [3]. 

Options for management of his hip dislocation were limited; a hip replacement was not an option due to the chronic dysplastic changes coupled with the hip girdle weakness. The Girdlestone procedure provided significant pain relief and allowed him to ambulate with a walker. As expected, there was no evidence of malignancy in the hip pathological specimen. Understanding the nature of plexiform neurofibromas and the potential tissue changes associated with them is important for clinicians treating patients who harbor them. In this case, the management was optimized via close collaboration with the multidisciplinary neurofibromatosis and sarcoma treatment teams. 

## Conclusions

In conclusion, we present the case of a 27-year-old male with NF1 that was followed for years with a pelvic girdle plexiform neurofibroma whose course was complicated by transformation to MPNST and a spontaneous hip dislocation. He underwent excision, Girdlestone procedure, chemotherapy, and radiation. It is critical to be aware of the potential of neurofibroma to malignantly transform and cause further pathology, such as hip dislocation. Finally, a multidisciplinary team is crucial to optimize patient management.

## References

[1] Huson SM, Harper PS, Compston DA (1988). Von Recklinghausen neurofibromatosis. A clinical and population study in south-east Wales. Brain.

[2] Lammert M, Friedman JM, Kluwe L, Mautner VF (2005). Prevalence of neurofibromatosis 1 in German children at elementary school enrollment. Arch Dermatol.

[3] Ruggieri M, Packer RJ (2001). Why do benign astrocytomas become malignant in NF1?. Neurology.

[4] Rutkowski JL, Wu K, Gutmann DH, Boyer PJ, Legius E (2000). Genetic and cellular defects contributing to benign tumor formation in neurofibromatosis type 1. Hum Mol Genet.

[5] Ly KI, Blakeley JO (2019). The diagnosis and management of neurofibromatosis type 1. Med Clin North Am.

[6] Beert E, Brems H, Daniëls B (2011). Atypical neurofibromas in neurofibromatosis type 1 are premalignant tumors. Genes Chromosomes Cancer.

[7] Plotkin SR, Bredella MA, Cai W (2012). Quantitative assessment of whole-body tumor burden in adult patients with neurofibromatosis. PLoS One.

[8] Evans DG, Baser ME, McGaughran J, Sharif S, Howard E, Moran A (2002). Malignant peripheral nerve sheath tumours in neurofibromatosis 1. J Med Genet.

[9] Nguyen R, Dombi E, Widemann BC (2012). Growth dynamics of plexiform neurofibromas: a retrospective cohort study of 201 patients with neurofibromatosis 1. Orphanet J Rare Dis.

[10] Rafaelian O, Coakley FV, Qayyum A (2006). Magnetic resonance imaging of hepatocellular carcinoma mimicking focal nodular hyperplasia: a potential pitfall in patients with cirrhosis?. J Comput Assist Tomogr.

[11] Galbraith JG, Butler JS, Harty JA (2011). Recurrent spontaneous hip dislocation in a patient with neurofibromatosis type 1: a case report. J Med Case Rep.

[12] Guilleminet M, Creyssel J, de Mourgues G, Fischer L (1970). [Von Recklinghausen's neurofibromatosis. Congenital hypertrophy of the lower limb in childhood and spontaneous luxation of the homolateral hip in adult age]. Presse Med.

[13] Haga N, Nakamura S, Taniguchi K, Iwaya T (1994). Pathologic dislocation of the hip in von Recklinghausen's disease: a report of two cases. J Pediatr Orthop.

[14] Kumar R, dos Reis Teixeira Neto A, Deavers MT, Amini B, Lewis VO (2014). Spontaneous hip dislocation secondary to intraarticular neurofibroma: a case report. Skeletal Radiol.

[15] Lachiewicz PF, Salvati EA, Hely D, Ghelman B (1983). Pathological dislocation of the hip in neurofibromatosis. A case report. J Bone Joint Surg Am.

[16] Waheed W, Diego F Lemos DF, Nathaniel Nelms N, Tandan R (2016). Multifactorial pathological hip subluxation in neurofibromatosis type-1 (NF1) due to intra-articular plexiform neurofibroma, lumbar radiculopathy and neurofibromatous polyneuropathy. BMJ Case Rep.

